# Statistical modeling of value distributions of similarity coefficients in virtual screening and its application to predicting fingerprint search performance

**DOI:** 10.1186/1758-2946-5-S1-O5

**Published:** 2013-03-22

**Authors:** Martin Vogt, Jürgen Bajorath

**Affiliations:** 1 Department of Life Science Informatics, B-IT, LIMES Program Unit Chemical Biology and Medicinal Chemistry, Rheinische Friedrich-Wilhelms-Universität Bonn, Bonn, 53113, Germany

## 

Similarity searching using fingerprints is a popular ligand-based virtual screening approach. The Tanimoto coefficient (Tc) is the most widely used measure for quantifying fingerprint similarity. In general, it is very difficult to assess the significance of the similarity of two molecules solely based on their calculated Tc values. In the literature, Tc cut-off values are frequently intuitively chosen as similarity criteria for virtual screening. This can be very problematic because the distribution of similarity scores largely depends on the specific type of fingerprint that is used and the reference compound for which the fingerprint is calculated. In order to rationalize similarity value considerations, a statistical approach named the *conditional correlated Bernoulli model *is presented that models similarity scores based on the statistical distribution of fingerprint features in large compound databases. Fingerprint features are modeled as dependent Bernoulli variables and conditional distributions of Tanimoto similarity values of database compounds are determined with respect to given reference compounds. The model makes it possible to estimate the position of a compound in a database ranking only based on its Tc value relative to the reference. This rank estimation of molecules enables the quantitative comparison of similarity values of different fingerprint types. Moreover, it can be utilized to rapidly assess the potential of fingerprints to identify new active molecules in a database search given a set of known reference molecules [1].
